# Knowledge, Attitudes and Practices among Women of Reproductive Age on Human Papillomavirus Infection, Cervical Cancer and Vaccination in Otukpo, Nigeria

**DOI:** 10.4314/ejhs.v34i1.3

**Published:** 2024-01

**Authors:** Olajide Akinnibosun, Onyukwo Grace Abakpa, Adole John Ujoh, Dominic Agbo Oche, Suleiman Zakari, Doowuese Yandev, Peter Adikwu, Onyemowo Okewu David, Oludare Agboola, Simon Paul, Onyemocho Audu, Emmanuel Odu, Innocent Achanya O Ujah, Joseph Anejo-Okopi

**Affiliations:** 1 Department of Microbiology, Federal University of Health Sciences, Nigeria; 2 Department of Biochemistry, Federal University of Health Sciences, Nigeria; 3 Department of Biological Sciences, Federal University of Health Sciences, Nigeria; 4 Department of Community Medicine, College of Medicine, Federal University of Health Sciences, Nigeria; 5 Institute of Global Health and Health Security, Federal University of Health Sciences, Nigeria; 6 Office of the Vice-Chancellor, Federal University of Health Sciences, Nigeria; 7 UNESCO International Centre for Biotechnology, University of Nigeria, Nigeria

**Keywords:** Human papillomavirus, Attitudes, Practices, Reproductive age, Vaccination, Otukpo, Nigeria

## Abstract

**Background:**

Knowledge, attitudes and practices (KAP) of human papilloma virus (HPV) is a necessary measure in curtailing delayed diagnosis and poor control practices. The objective of this study was to assess the knowledge, attitudes and practices vis-à-vis HPV infection, cervical cancer and vaccination among women.

**Methods:**

This cross-sectional study was conducted at 15 selected health-care facilities in Otukpo metropolis and it involved 168 pregnant women. The data were collected using structured questionnaire, and analysed for descriptive and analytical statistics using Epi Data Version 3.1 and SPSS statistical package Version 21.

**Results:**

Most of the respondents (75.0%) have heard of human papilloma virus and their information source were mostly the health-care providers. In total, 132(78.6%) agreed to take the vaccine if offered for free but 152(90.5%) stated that it is imperative to seek the opinion of health providers before vaccine uptake. However, only 27(16.1%) have undergone recommended checkup for human papilloma virus/cervical cancer and 23(13.7%) have taken at least a vaccine dose. Some respondents 66(39.3%) had good knowledge while 95(56.6%) demonstrated positive attitude. However, most respondents 161(95.8%) demonstrated poor practices.

**Conclusions:**

There is enormous need to improve HPV sensitization especially in women due to cervical cancer associated risks. Healthcare personnel are therefore encouraged to create more awareness on HPV infection and screening of cervical cancer (CC) via counseling sessions and communications tool like the new media. KAP approach is a critical tool towards successful CC screening and HPV control.

## Introduction

Human papilloma viras (HPV) is a viral infection of global importance and a prevalence that varies widely across geographical regions. Notably, sub-Saharan Africa remains a region that is prominently threatened by HPV (1). HPV infection is mainly transmitted via sexual contact (2), and most sexually acquired cases of HPV usually occurred shortly after becoming sexually active (3). According to global reports, 75% of sexually active individuals got infected with HPV infection over their lifespan (4).

In many developing nations of the world, women especially those of parturition age face umpteen social and health challenges. In Nigeria, about 23.7% of women and 73% of men contract HPV genital infection within the first three years of sexual debut (5). HPV infection has been indicated to be pervasive among juvenile women of age 16-22 years (6). Most times, HPV infection can be transient, latent and unnoticed for a prolonged period due to the activities of antibodies (7). However, chronic high-risk HPV infection has been severally linked to the growth of precancerous cells which subsequently triggers the development of cervical cancer (CC) (2).

Globally, cervical cancer is the fourth most common cancer in women with about 604,000 new cases and 342,000 deaths in 2020, and about 90% of the new cases and deaths in 2020 occurred in low- and middle-income countries (8). In Nigeria, cervical cancer ranks as the second most common cancer in women aged 15-44 years with about 14,943 new cases diagnosed annually (5). HPV cases are expected to intensify to about 9.9 million with 5.5 million deaths annually by 2030 as the world population increases (9).

Vaccination, cytological examination and health sensitization represent the main strategies for HPV prevention and control (10). These control strategies against cervical cancer are enhanced by early detection of HPV and the treatment of pre-cancerous lesions. The HPV vaccine has also been recommended by WHO as the fundamental approach for the prevention of cervical cancer, and should ideally be administered preceding first sexual contact (11). Although the vaccines have been approved for administration in Nigeria since 2009, it is mostly accessible to privileged individuals that can afford the cost (11). This contributed to the major challenges limiting HPV vaccination exercise in Sub-Saharan Africa which is the non-release of funds by government agencies to sponsor free vaccination and the inability of low-earners to self-sponsor (12-14). Presently in Nigeria, HPV vaccines are mostly available in private health facilities and at unreadily affordable rates. Despite the impending danger this could result into, the efforts being made by the Nigerian government to incorporate HPV vaccine into the national basic immunization programme remain rather slow (3). However, several African countries have started having access to HPV vaccine through the assistance of Global Alliance for Vaccines and Immunization (GAVI) and Nigeria is currently in the pre-introduction era of the vaccine uptake among adolescent females (15,16).

Nevertheless, the understanding of the implications of HPV infection and responses towards public acceptability of the vaccine have been low as several individuals demonstrated hesitation on getting administered even when free (13,16). Vaccine safety is a major concern expressed by the individuals who were reluctant to accept the vaccination of especially in teenage females (17). This study aimed to assess the knowledge, attitudes, and practices of HPV infection among women of reproductive age as it relates to vaccination and cervical cancer awareness in Otukpo, Benue State, Nigeria.

## Materials and Methods

**Study design**: The descriptive cross-sectional study was conducted in fifteen health facilities in Otukpo Township. Otukpo local government area has an estimated population of 266,411 in which 129,799 is females (18). The study was conducted between November and December 2021 using structured questionnaire which was adapted in accordance with previous literatures (19-21). The variables analyzed include socio-demographic information, knowledge of HPV, attitude and practice towards HPV.

**Sample size**: This study involved 168 women that were selected for the study from the 15 health facilities that provides maternity care services in Otukpo metropolis based on the population of women registered for antenatal checkup during the study period- The inclusion criteria include consenting pregnant women visiting the health facilities for antenatal check-up at the time of conducting the research. Participants that declined consent were excluded. The sample size (n) was calculated using the Taro-Yamane formula $n=\frac{N}{\left(1+N\;(e)2\right)}$ at 95% confidence interval as previously described (22,23). Where N= Total population under study and e= 5% minimal error. Based on the health facilities' records, the total population of women registered for antenatal checkup during the study period was 284, which connoted the sample size to be a minimum of 166 participants.

**Sampling procedure and data collection**: Structured questionnaire adapted from a previous study was administered for data collection. Information was obtained from the respondents using structured questionnaire adapted from previous studies (19-21). The questionnaires were written in English language and interpreted in local dialects upon request. The questionnaire included the respondents' socio-demographic characteristics, and their perspectives as regards the knowledge, attitude and practice of human papilloma virus infection, mode of transmission, signs and symptoms, treatment and preventive measures. The composite measure of respondents' knowledge, attitude and practice was measured using a scoring system where values were assigned to positive response in knowledge, attitude or practice section, while zero score was awarded for a negative response. Positive response of 80% and above was considered good, positive response greater than 50% but less than 80% was considered fair and positive response of 50% or less was classified as poor. In knowledge, 9-11 (≥80%) positive response was considered as good, 6-11 (50%-79%) was considered as fair while ≤5 (≤50%) was classified as poor knowledge. In attitude, 6-7 (≥80%) positive response was considered as good, 4-5 (50%-79%) was considered as fair while ≤3 (≤50%) was classified as poor knowledge. In practice, 4-5 (≥80%) positive response was considered as good, 3 (50%-79%) was considered as fair while ≤2 (≤50%) was classified as poor knowledge.

**Statistical analysis**: The data was analyzed using Epi Data Version 3.1 and SPSS statistical package Version 21. Descriptive statistics and cross tabulations were used to explore statistical relationships between the study population and relevant variables. Multivariate logistic regression models were used in determining factors associated with the dependent variables and independent variables. Odds ratio was determined using 95% Confidence Intervals (95% CI). Chi square test was used to explore proportional relationship between groups. The level of statistical significance was at p < 0.05 (providing 95% confidence interval).

**Ethics**: The research protocol was designed according to the guidelines recommended for biomedical researcher involving Human Subjects and the ethical standards of the declaration of Helsinki and reviewed by the Health Research Ethics Committee of Federal University of Health Sciences, Otukpo. The research protocols was approved by the Health Research Ethics Committee (HREC) General Hospital, Otukpo, Benue State with Reference no: GHOB/HREC/26-10-2021-006 as it pose low and negligible risks to participants. The respondents were acquainted with adequate information regarding the study before the interviews were conducted and confidentiality of the respondents was ensured. Informed consent was obtained from all participants as regards this study. The management of hospitals and health facilities gave permission to prior to data collection.

## Results

**Socio-demographic characteristics among women of reproductive age towards HPV**: A total of 168 pregnant female participated in this study. The socio-demographic details of the participants showed that ages of the respondents ranged from 18 to 57 years with the mean age at 25.41 ± 7.26 years. Majority of the respondents 96(57.1%) has attained primary and/or secondary education, 94(56.0%) have no child yet while 81(48.2%) were primigravid.

**Knowledge of human papilloma viras among women of reproductive age**: Most of the respondents 126(75.0%) have previously heard of human papilloma virus and the main source of their information was healthcare providers (74.6%). However, only 105(62.5%) were aware that HPV can be transmitted sexually. A total of 85(50.6%) were aware that HPV could cause cervical cancer while 119(70.8%) affirmed that HPV vaccine could protect them from getting infected.

**Attitude of women of reproductive age to the human papilloma virus infection**: With regard to the respondents' attitudes to HPV, 108(64.3%) agreed that HPV could trigger other health complications. In total, 132(78.6%) agreed to take the vaccine if offered for free and 152(90.5%) stated that it is compulsory to seek the opinion of health providers before taking the HPV vaccine.

**Preventive practice of women of reproductive age to the human papilloma virus infection**: In terms of preventive approaches among the respondents, only 27(16.1%) have undergone recommended checkup for human papilloma virus/cervical cancer while 23(13.7%) have taken a preventive dose of the HPV vaccine. In total, 66(39.3%) affirmed that they use condoms during sex as singles.

**Relationship of factors associated with knowledge, attitude and practice towards HPV**: Using multivariate logistic regression, it was observed that age group (18-28) was 0.231 times likely to be knowledgeable on HPV infection than other age groups. Additionally it was observed that age group (18-28) was 0.925 times more likely to be exhibit good attitude towards the prevention of HPV compared to age group (29-30) while age group (18-28) were 0.436 likely to demonstrate better attitude towards HPV infection compared to other age groups. Although some of the respondents having good knowledge and attitude to HPV, most of them demonstrated very poor practice. The effect of the variables lacks significant association with knowledge, attitude and practices as the p-value were greater than 0.05, with few exceptions based on the number of children and pregnancy history.

## Discussion

Improved knowledge, attitudes and practices in respect to HPV infection and screening are practical approaches in mitigating the burden of cervical cancer among women. This study was conducted to assess the knowledge of women of reproductive age in Otukpo about the human papillomavirus.

Majority of the respondents (75.0%) have heard of human papilloma virus. This is relatively high and consistent with the 83.8% and 63.3% reported by comparable maternity clinic-based studies in Nigeria (24) and Russia (25) respectively. However, school-based studies on female reported lower awareness level of 0.9% and 30% in Nigeria (26,27) and 43.2% in South Africa (28). The disparity in awareness between maternity clinic-based and school-based studies could be attributed to public perspectives that adolescents and young female respondents in school-based studies are not matured enough for discussions relating to sexual activity as reported in previous studies (29,30). A study on primigravid antenatal clinic attendees earlier reported that only 5.4% of the respondents have heard of HPV on their first antenatal visit (31). Higher level of awareness in maternity/clinic-based studies further attest to the influence healthcare workers exert on disease awareness and general health information management. Profound public conviction conferred on health care workers as reliable sources of health information has been widely reported (32,33).

In this study, majority of the participants (62.5%) were aware that HPV can be transmitted via sex. Higher level of awareness (87.0% and 78.0%) that HPV can be transmitted via sexual intercourse have been reported in comparable clinic-based studies in Nigeria (19,34). Contrarily, very low level of awareness (0.9%) regarding the sexual transmission of HPV has been reported among adolescent school children in Nigeria (27). Most of the respondents (60.7%) in this study considered the use of condom a practice that is not mandatory in the prevention of HPV. In disagreement to this, frequent use of condoms has been notably associated with dwindling rates of sexually transmitted infections including HPV infection (35).

Among the respondents, 50.6% were aware that HPV could cause cervical cancer while 70.8% affirmed that HPV vaccine could prevent infection. Contrarily, our finding is significantly higher than the 8%, 8.8% and 29.5% reportedly aware that HPV could cause cervical cancer in previous studies among women of reproductive age in Gabon, Ethiopia and Nigeria respectively (20,21,36). Inadequate awareness on cervical cancer has also been reported in middle-income and high-income countries (29,37,38). This implies that the low awareness on cervical cancer is a global situation especially among adolescents and young adults. Low level of awareness on HPV and its correlation with cervical cancer is a major factor behind the unwavering burden of cervical cancer and other HPV related diseases (36).

In this study, most of the respondents (67.3%) positively responded that all sexually active females should be given the HPV vaccine before sex debut. In Nigeria, the relatively expensive nature of the vaccine adversely affects its uptake (19), despite its recommendation by the Nigerian federal ministry of health (39). However, parent and guardian are usually reluctant on early vaccination and discussions on sexually related infections with the younger age groups because it may give them impressions that they are now matured enough to get involved in sexual activities (30,40). Most of the respondents (90.5%) in this study emphasized that it is compulsory to seek information from health practitioners before taking HPV vaccine while some stated that they would refuse taking the vaccine even when offered for free. This agrees with reports from earlier studies among women in Europe and America that reasons for the unwillingness in getting vaccinated include concerns of vaccine safety and the demand for health workers opinion other than financial costs (29,41).

The knowledge and practice of Pap smear test from this study was relatively poor as more than half of the respondents do understand its relationship with the detection of HPV infections and only 4.2% of the total respondents have taken the test. This is similar to findings from studies conducted in developing countries (42,43) but differs from the studies conducted in developed countries (44,45). The variance in level of awareness, practice and acceptability of cervical screening could be due to factors such as illiteracy, poverty, ignorance and ineffective cervical cancer screening program (46).

In this study, it was observed that age group [29-42] was 0.383 times likely to have more knowledgeable about HPV infection than [18-28] age group. Previous study has encouraged women at all ages to seek information and regularly get screened for HPV related infection including cervical cancer (47). Furthermore, it was observed in this study that participants that have attained higher education were more likely to have more knowledge on HPV related infections than those that haven't. This is an indication that educated women generally have more access to information on HPV infection and its vaccination as previously reported in a study among women in Nigeria (48). Also, it has been reported that women with higher education and those with children were more likely to be knowledgeable about HPV in comparison to women with lower educational and the nonparturitive women in a maternity clinic-based study in Russia (25). However, the respondents that demonstrated good knowledge and attitude towards HPV observably demonstrated very poor practice. This agrees with the findings from previous study among women in a clinic-based survey in Nigeria that although better education is a prominent tool in health promotion, it isn't the sole determinant of better health practices (47). Therefore, as educational efforts to improve the knowledge on HPV is on the increase, there are need to further inform the people and health workers on the importance of improved practice as it is a major tool towards HPV prevention.

In conclusion, the findings of this study highlight the need to improve the knowledge of HPV among younger age groups and regular screening among pregnant women. HPV vaccination has a relatively low coverage in Nigeria and the low awareness of HPV vaccine revealed in this study further emphasize on the need to improve HPV awareness and acceptability by including the vaccine in the national immunization routine schedule. HPV infection remains critical to reproductive women and they need to be properly informed about the merits of vaccination and the health threats associated with not receiving the vaccine. This is due to the fact that poor understanding as regards the dangers of HPV and its consequences could result in permanent health difficulties including cervical cancer. Therefore, intense health campaign on HPV and HPV vaccine among women of reproductive age is notably recommended.

## Figures and Tables

**Table 1:** Distribution socio-demographic characteristics among women of reproductive age

Characteristics	Frequency	Percentage (%)
**Age (in years)**		
18 – 28	122	72.6
29 – 42	41	24.4
43 – 57	5	3.0
**Habitat**		
Rural	88	52.4
Urban	80	47.6
**Person living with**		
Alone	28	17.7
Husband	70	41.7
Boyfriend	3	1.8
Friends	7	4.2
Relatives	60	35.7
**Level of education**		
No formal education	0	0
Primary	3	1.8
Secondary	93	55.4
Higher education	72	42.9
**Religion**		
Christian	158	94.0
Muslim	10	6.0
**Marital status**		
Single	89	53.0
Married	79	47.0
**Occupation status**		
Self employed	90	53.6
Govt, employed	18	10.7
Unemployed	60	35.7
**Previous history of pregnancy**		
None	81	48.2
1 – 4	68	40.5
5 – 8	18	10.7
9 – 11	1	0.6

**Table 2:** The knowledge of human papilloma virus among women of reproductive age

Knowledge items	Response	Frequency	Percent
Have you heard of human papilloma virus?	Yes	126	75.0
	No	42	25.0
If Yes, what is your source of information?	Radio/TV	29	23.0
	Healthcare provider	94	74.6
What causes HPV infection?	Virus	111	66.0
	Bacteria	20	11.9
	Fungi	5	3.0
	I don't know	32	19.1

**Figure 1:**
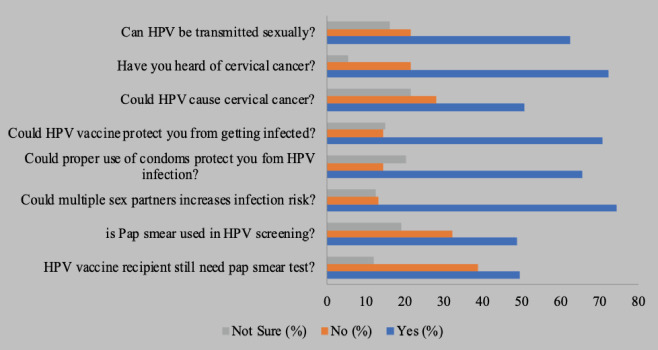
Human papilloma virus knowledge among women of reproductive age

**Figure 2:**
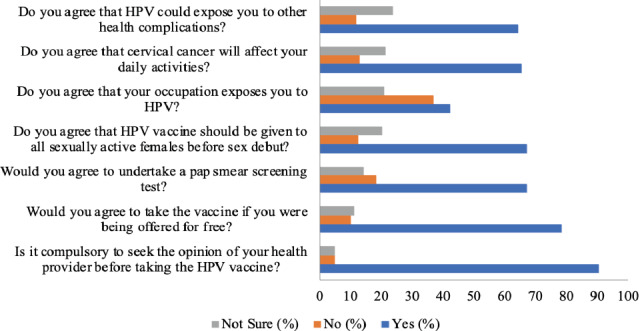
The attitude of women of reproductive age towards human papilloma virus

**Figure 3:**
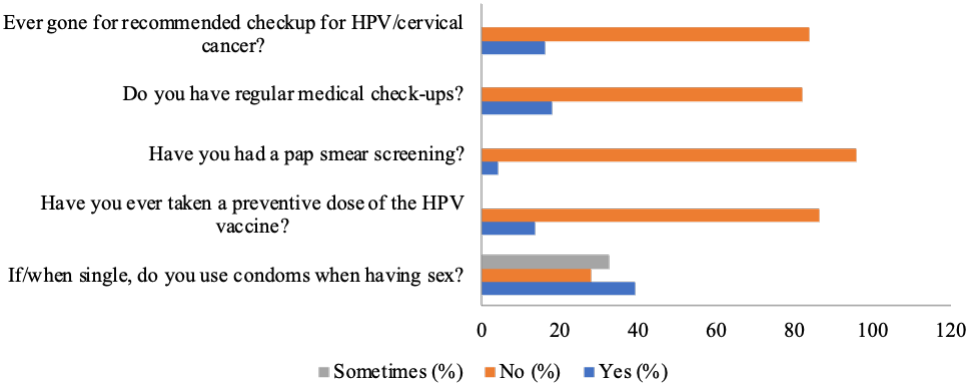
The preventive practice of women of reproductive age towards human papilloma virus

**Table 3:** Relationship of factors associated with knowledge, attitude and practice towards HPV

VARIABLE	Knowledge		AOR [95% CI]	P-Value	Attitude		AOR [95% CI]	P-Value	Practice		AOR [95% CI]	P-Value
	Good (%)	Poor (%)			Good (%)	Poor (%)			Good (%)	Poor (%)		
**Age**												
18 – 28	41 (33.6)	52 (42.6)	0.231	0.134	68 (55.7)	21 (17.2)	0.925	0.941	3 (2.5)	117 (95.9)	0.436	0.228
			[0.03-1.6]				[0.1-7.2]				[0.1-1.7]	
29 – 42	21 (51.2)	12 (29.3)	0.383	0.332	23 (56.1)	5 (12.2)	0.777	0.812	0 (0)	40 (97.6)	0.219	0.137
			[0.06-2.7]				[0.1-6.2]				[0.03-1.6]	
43 – 57	4 (80.0)	0 (0)			4 (80)	0 (0)			1 (20.0)	4 (80.0)		
**Habitat**												
Rural	33 (37.5)	29 (33.0)	1.073	0.731	50 (56.8)	12 (13.6)	0.989	0.963	4 (4.5)	83 (94.3)	2.356	0.178
			[0.7-1.6]				[0.6-1.6]				[0.7-8.2]	
Urban	33 (41.3)	35 (43.8)			45 (56.3)	14 (17.5)			0 (0)	78 (97.5)		
**Level of education**												
Primary	1 (33.3)	1 (33.3)			1 (33.3)	0 (0)			0 (0)	3 (100)		
Secondary	33 (35.5)	38 (40.9)	0.780	0.755	55 (59.1)	11 (11.8)	1.438	0.690	3 (3.2)	90 (96.8)	242328.71	0.000
			[0.2-3.7]				[0.2-8.6]					
Higher education	32 (44.4)	25 (34.7)	1.189	0.829	39 (54.2)	15 (20.8)	0.854	0.863	1 (1.4)	68 (94.4)	271971.15	
			[0.3-5.7]				[0.1-5.1]					
**Religion**												
Christian	62 (39.2)	60 (38.0)	0.798	0.614	90 (57.0)	23 (14.6)	1.587	0.344	4 (2.5)	151 (95.6)	4560169.98	
			[0.3-1.9]				[0.6-1.1]					
Muslim	4 (40.0)	4 (40.0)			5 (50)	3 (30)			0 (0)	10 (100)		
**Marital status**												
Single	39 (43.8)	28 (31.5)	1.227	0.318	56 (62.9)	11 (12.4)	1.271	0.328	3 (3.4)	85 (95.5)	1.136	0.797
			[0.8-1.8]				[0.8-2.1]				[0.4-3.0]	
Married	27 (34.2)	36 (45.6)			39 (49.4)	15 (19.0)			1 (1.3)	76 (96.2)		
**Occupation status**												
Self employed	34 (37.8)	37 (41.1)	0.830	0.402	49 (54.4)	13 (14.4)	1.039	0.887	1 (1.1)	88 (97.8)	0.480	0.187
			[0.5-1.3]				[0.6-1.8]				[0.2-1.4]	
Govt, employed	6 (33.3)	8 (44.4)	0.819	0.577	9 (50.0)	4 (22.2)	0.833	0.657	0 (0)	17 (94.4)	0.722	0.700
			[0.4-1.7]				[0.4-1.9]				[0.1-3.8]	
Unemployed	26 (43.3)	19 (31.7)			37 (61.7)	9 (15.0)			3 (5.0)	56 (93.3)		
**Number of children**												
None	39 (41.5)	31 (33.0)	1.350	0.182	57 (60.6)	13 (13.8)	1.167	0.545	3 (3.2)	90 (95.7)	8535920.35	0.000
			[0.9-2.1]				[0.7-1.9]					
1 – 4	18 (30.5)	30 (50.8)			27 (45.8)	12 (20.3)			0 (0)	59 (100)		
5 – 8	9 (60.0)	3 (20.0)	1.912	0.114	11 (73.3)	1 (6.7)	1.341	0.581	1 (6.7)	12 (80.0)	17267984.95	
			[0.9-1.3]				[0.5-3.8]					
**History of pregnancy**												
None	37 (45.7)	23 (28.4)	0.800	0.535	52 (64.2)	11 (13.6)	0.817	0.676	3 (3.7)	78 (96.3)	0.659	0.461
			[0.4-1.6]				[0.3-2.1]				[0.2-2.0]	
1 – 4	17 (25.0)	37 (54.4)	0.461	0.038	29 (42.6)	14 (20.6)	0.688	0.442	0 (0)	67 (98.5)	0.270	0.182
			[0.2-1.0]				[0.3-1.8]				[0.04-1.9]	
5 – 8	11 (61.1)	4 (22.2)			13 (72.2)	1 (5.6)			1 (5.6)	16 (88.9)		
9 – 11	1 (100)	0 (0)	1798957.9		1 (100)	0 (0)	1753955.2		0 (0)	0 (0)	2.931	0.350
											[0.3-28.0]	
